# Osseous variations associated with physiological thinning of the glenoid articular cartilage: an osteological study with CT, MRI and arthroscopic correlations

**DOI:** 10.1007/s00256-023-04358-9

**Published:** 2023-05-25

**Authors:** Michal Benes, Petr Fulin, David Kachlik, Azzat Al-Redouan, Jan Tomaides, Martin Kysilko, Sarka Salavova, Vojtech Kunc

**Affiliations:** 1 Department of Anatomy, Second Faculty of Medicine, Charles University, Plzenska 130/221, 150 06, Prague 5, Czech Republic; 2 Center for Endoscopic, Surgical and Clinical Anatomy (CESKA), Second Faculty of Medicine, Charles University, Prague, Czech Republic; 3 1st Department of Orthopaedics, First Faculty of Medicine, Charles University and University Hospital Motol, Prague, Czech Republic; 4 Department of Health Care Studies, College of Polytechnics, Jihlava, Czech Republic; 5 Department of Radiology, Second Faculty of Medicine, Charles University and University Hospital Motol, Prague, Czech Republic; 6 Clinic of Trauma Surgery, Masaryk Hospital, Usti Nad Labem, Czech Republic

**Keywords:** Bare spot, Bare area, Tubercle of Assaky, Intraglenoid tubercle, Glenoid fovea, Terminologia Anatomica

## Abstract

**Objective:**

To investigate the relationship between osseous variations of the glenoid fossa and thinning of the overlaying articular cartilage.

**Materials and methods:**

In total, 360 dry scapulae, comprising adult, children and fetal specimens, were observed for potential presence of osseous variants inside the glenoid fossa. Subsequently, the appearance of the observed variants was evaluated using CT and MRI (each 300 scans), and in-time arthroscopic findings (20 procedures). New terminology of the observed variants was proposed by an expert panel formed by orthopaedic surgeons, anatomists and radiologists.

**Results:**

Tubercle of Assaky was observed in 140 (46.7%) adult scapulae, and an innominate osseous depression was identified in 27 (9.0%) adult scapulae. Upon radiological imaging, the tubercle of Assaky was found in 128 (42.7%) CTs and 118 (39.3%) MRIs, while the depression was identified in 12 (4.0%) CTs and 14 (4.7%) MRIs. Articular cartilage above the osseous variations appeared relatively thinner and in several young individuals was found completely absent. Moreover, the tubercle of Assaky featured an increasing prevalence with aging, while the osseous depression develops in the second decade. Macroscopic articular cartilage thinning was identified in 11 (55.0%) arthroscopies. Consequently, four new terms were invented to describe the presented findings.

**Conclusion:**

Physiological articular cartilage thinning occurs due to the presence of the intraglenoid tubercle or the glenoid fovea. In teenagers, the cartilage above the glenoid fovea may be naturally absent. Screening for these variations increases the diagnostic accuracy of glenoid defects. In addition, implementing the proposed terminological updates would optimize communication accuracy.

## Introduction

Defects of the bony glenoid and adjacent articular cartilage are known to be a predisposing factor for recurrent dislocations of the shoulder [1, 2]. However, not all osteocartilaginous irregularities necessarily refer to pathological conditions or predispose to shoulder instability. Emphasis should be placed on profound understanding of surgical anatomy to avoid its misinterpretation and reach a correct diagnosis. Specifically, the tubercle of Assaky together with the bare spot was reported to be normal anatomical findings affecting the glenoid fossa that should not be confused with osteocartilaginous defects [3].

The tubercle of Assaky was originally described as a small bony protrusion located just below the centre of the glenoid fossa [4]. The hyaline cartilage overlaying the tubercle of Assaky hence appeared thinned and macroscopically translucent, presumably due to the thickened subchondral bone [4]. This cartilage thinning was then observed by several other authors and is nowadays termed the bare spot or bare area [1, 2, 5-13]. The bare spot was reported to be a common finding among all age groups [14]. Nevertheless, it has also been noted that the original studies used different and not unified criteria [6]. Some authors considered the bare spot as a thinning of the hyaline cartilage irrespective of its underlying bone texture [1, 5-10, 12, 15], while others define it as a reactive cartilage loss secondary to the presence of the tubercle of Assaky [13, 16], or as a focal cartilage thinning or eventual absence due to a defect in the subchondral bone [11, 14, 17].

The abovementioned morphological entities have been studied intraoperatively [1, 2, 7, 11-13, 18], radiologically with the use of MRI and CT [2, 10-14, 16, 17], or with traditional anatomical dissections [1, 5, 6, 8-10, 15, 16]. Nevertheless, none of the existing studies presents a complex investigation that focuses on the causes of articular cartilage thinning. To our best knowledge, no study to date has evaluated the osseous variations using solely dry scapulae that could potentially bring new insights into the articular surface anatomy of the glenoid.

This study aims to investigate, through osteological observations of dry scapulae, radiological imaging methods and arthroscopy of the shoulder joint, the osteocartilaginous variations of the glenoid fossa referred to as a bare spot. Furthermore, we aim to elucidate their developmental aspects and to clarify the nomenclature by proposing new anatomical terms based on an expert consensus.

## Materials and methods

The concept of this study was designed in accordance with the ethical standards established in the 1964 Declaration of Helsinki and its later amendments, and the study protocol was approved by the Ethics Committee for Multi-Centric Clinical Trials of the University Hospital Motol and Second Faculty of Medicine, Charles University in Prague (EK-1107/22).

### Anatomical part using dry scapulae

For the anatomical part of the presented study, a total of 360 dry scapulae from the collections of First, Second and Third Faculty of Medicine, and Faculty of Medicine in Hradec Kralove, Charles University, Czech Republic, were investigated for the presence of morphological variations inside the glenoid fossa. The sample was composed of 300 adult scapulae (149 right, 151 left) of unknown sex and age at the time of death, 30 children scapulae (11 right, 19 left) of unknown sex and estimated age between 1 and 10 years, and 30 scapulae belonging to fetuses (16 right, 14 left) of unknown sex and estimated age between 30 and 40 weeks of intrauterine development. Only intact bones without any visible degenerative or other pathological changes were deemed eligible for inclusion in this study.

Once an osseous variation was visually identified inside the glenoid fossa, further morphometric parameters were studied. The height or depth was measured using a digital tread depth gauge (Aideepen, China) with an accuracy of 0.01 mm specified by the manufacturer. After that, the glenoid fossa was photographed alongside a scale tape and the pictures were then imported into the image analysis software Fiji ImageJ v.2.0.0 (National Institutes of Health, USA), where the antero-posterior extent (considered as the width) and the supero-inferior extent (considered as the length) were acquired using a ‘straight tool’ calibrated from the known distance on the tape. Furthermore, the ‘freehand selection tool’ was used to measure the area of the osseous variations and this value was then compared with the area of the whole glenoid fossa. To provide the exact locations of the studied structures within the glenoid fossa, a Cartesian coordinate system in a plane was constructed [19]. Two lines were drawn in the photographed glenoid fossa where the vertical line ran above the supraglenoid and infraglenoid tubercles in continuity with the long axis of the glenoid, and the horizontal line was placed in the middle of the length of the glenoid fossa perpendicular to the vertical line. These vertical and horizontal lines served as the *x*-axis and *y*-axis for the coordinate system. To standardise the various shapes and sizes of the glenoid fossa, its length and width detected from the computer-assisted image analysis software were averaged in order to be transferred to a unified plane. Normalisation of the coordinates in regard to the location of the osseous variations was achieved by percentage by adding or deducting the difference between the individual and averaged size of the glenoid fossa to the *x*-axis and *y*-axis. The coordinates were acquired overlaying a ‘grid’ with a scale set at 1 mm. Consequently, the probability of finding the distinct osseous variation within an area of 1 × 1 mm was graphically expressed as percentage in 10% tiers and a heatmap was constructed [20].

### Radiological part using CT and MRI scans

The radiological part of the presented study included a total of 300 CT and 300 MRI scans of the glenohumeral joint retrieved from the database of the Department of Radiology, Second Faculty of Medicine, Charles University and University Hospital Motol, Prague, Czech Republic to correlate the osteological findings with the appearance of the adjacent articular cartilage. To elucidate the developmental aspects of the aforementioned osseous variations, 50 scans from each of the following six age groups were randomly selected: (1) 1–10 years old; (2) 11–20 years old; (3) 21–30 years old; (4) 31–40 years old; (5) 41–50 years old; (6) 51 + years old. Overall, the sample was composed of 153 right and 147 left CT scans belonging to 198 male and 102 female patients, and 178 right and 122 left MRI scans belonging to 183 male and 117 female individuals. Patients with conditions that could interfere with the natural anatomy, namely, fractures of the glenoid, severe degenerations, shoulder replacements, tumorous affections of the scapula or poor scan quality, were not enrolled in this study.

The CT scans were obtained using Siemens SOMATOM Definition 7,740,769 (Siemens, Germany) and Toshiba Aquilion TSX 101-A (Toshiba, Japan) devices with slice thickness of 1.5 mm. Each joint was inspected in coronal, axial and sagittal planes. The MRI scans were acquired with the use of 1.5-T Philips Intera Achieva (Philips, Netherlands) and 1.5-T Siemens MAGNETOM Avanto (Siemens). A standardized protocol, consisting of PD/TSE sequence in three planes, T1/TSE sequence in parasagittal plane and T2/TSE sequence in paracoronal plane, was used with slicing thickness of 3.0 mm. For both imaging modalities, the xVision View v. 2.7.1. (Vidis, Czech Republic) was used to display the captured glenohumeral joints.

### Arthroscopic part

Arthroscopic evaluation of the glenoid fossa was carried in 20 patients undergoing procedures for rotator cuff repair. The cohort comprised 7 males and 13 females, with a mean age of 36.4 (range 19–54) years. All patients were treated by orthopaedic surgeons affiliated with the 1st Department of Orthopaedics, First Faculty of Medicine, Charles University and University Hospital Motol, Prague, Czech Republic. During the procedure, the lateral decubitus position of the patient was achieved with traction on the operated upper limb. Optical instruments Matrix E Spectar and Matrix LED duo (Xion Medical GmbH, Germany) with medical imaging recorder UR-4MD (TEAC Europe GmbH, Germany) were used. The glenohumeral joint was accessed from the anterior portal and the articular cartilage covering the glenoid fossa was inspected for any apparent signs of cartilage thinning, including irregularities in the shape or structure, and colour changes. To assess the underlaying osseous anatomy, the intraoperative findings were then compared with the preoperatively performed MRI.

### Terminology consensus

As a result of our findings from the anatomical, radiological and arthroscopic parts, new terminology proposals were made based on the consensus method of Delphi [21]. An expert panel, composed of recognised experts in orthopaedic surgery, anatomy and radiology, was invited to fill out an electronic questionnaire created in Google Forms (Google, USA). Multiple rounds survey was designed in order to reliably reach adequate agreement. In the first round, a preliminary list of terms proposed by the authors, including their descriptions and illustrative figures, was sent. The nominees were asked to rate their level of agreement with each proposed term on a scale from one (low) to five (high), and eventually to comment or contribute with their personal preferences. For the following rounds, the questionnaire was revised to reflect the reservations and suggestions from the previous round. Then, the same nominees were asked to evaluate the proposals on the same five-level scale. In all rounds, the scores for each term were averaged and the proportions were rated, according to a priori set intervals, as ‘oppose’ from 1 to < 2.5; ‘neutral’ from ≥ 2.5 to ≤ 3.5 and ‘concur’ from > 3.5 to 5. If a term scored > 3.5 (concur) in a particular round, it was considered as an established agreement and was therefore not further included in the following rounds.

### Quality assessment and statistical analysis

Reduction of the inter-observer variability was achieved by photographic digitalisation of the findings and subsequently reached consensus with other authors. To assess the intra-observer variability during the data curation, all measurements were taken three times and the average number was used for further analysis. All morphometric data are expressed as a mean value ± standard deviation (range). The chi-squared test of independence was used to evaluate the differences between categorical variables. The level of significance was set at *p* < 0.05. Linear regression model was used for investigating the relationship between input and output variables. Calculation of the required sample size for anatomical study was based on previous findings stating the prevalence of the bare spot around 75%. With the desired precision set at 0.05 and confidence level 95% with sensitivity and specificity values equal 0.999, the sample size should be at least 290. The statistical analysis was performed in Numbers v.11.2 (Apple, USA).

## Results

### Anatomical part

**Fig. 1:**
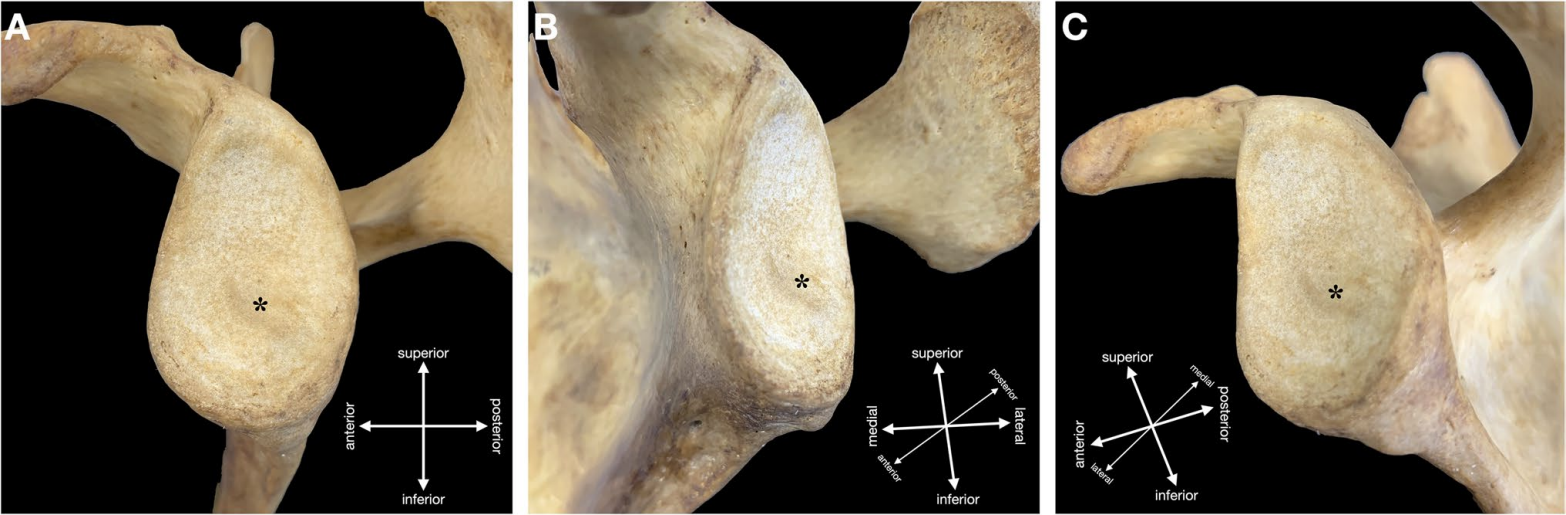
Left adult scapula containing the tubercle of Assaky (asterisk) inside the glenoid fossa. Captured from lateral (**A**), anterolateral (**B**) and posterolateral (**C**) views

**Fig. 2:**
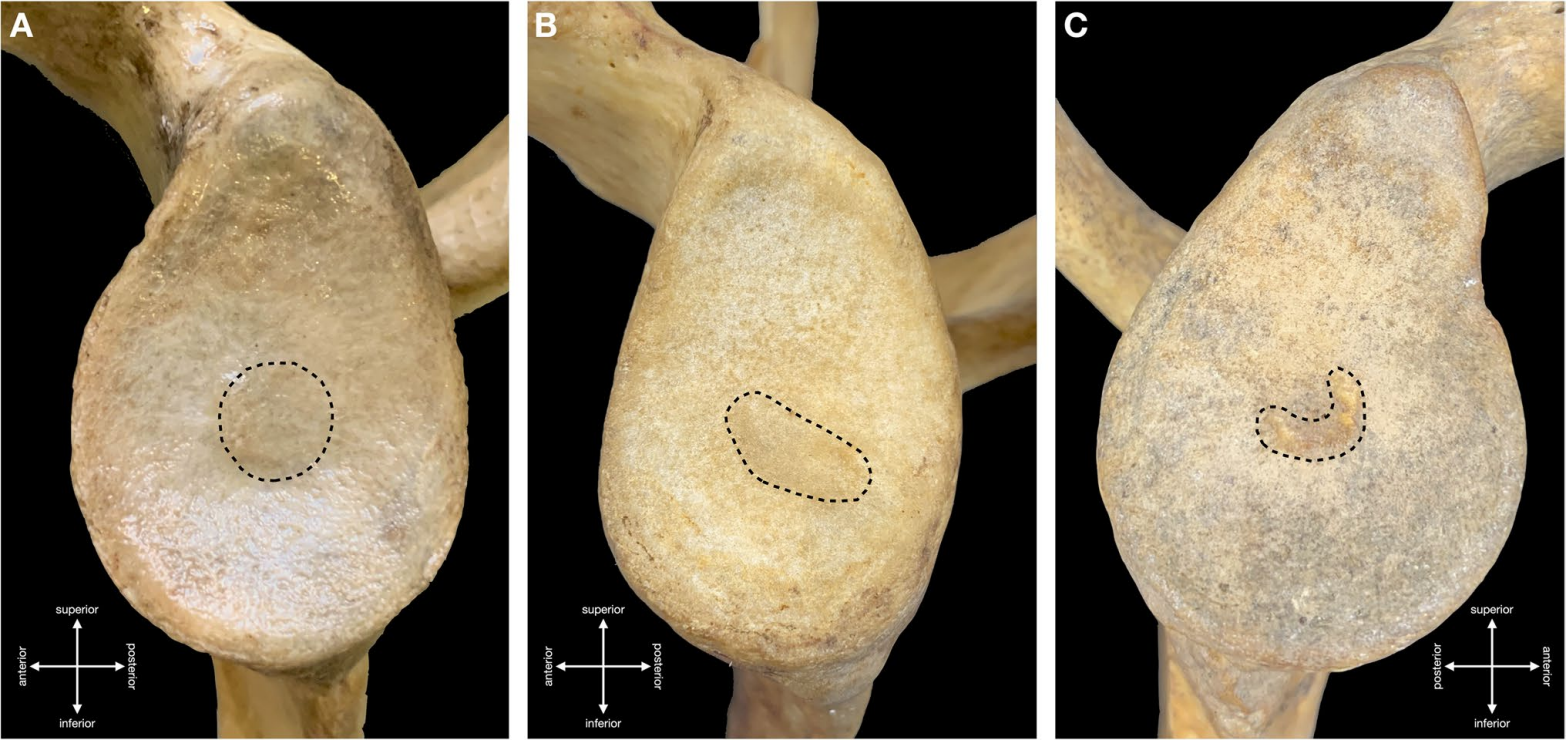
The tubercle of Assaky featuring three distinct shapes, such as oval (**A**), longitudinal (**B**) and sickle (**C**)

Out of the 300 adult scapulae, the tubercle of Assaky was present in 140 cases (46.7%) (Fig. 1). It was found in 64 right scapulae (43.0%) and in 76 left scapulae (50.3%) with no statistically significant difference between the sides (*p* = 0.423). Morphologically, the tubercle of Assaky featured three distinct shapes that were classified as (1) oval, which was present in 77 cases (55.0%); (2) longitudinal, which was observed in 49 cases (35.0%); and (3) in 14 cases (10.0%), it resembled a sickle (Fig. 2). The acquired measurements are shown in Table 1. The tubercle of Assaky occupied a mean area of 29.5 ± 13.2 mm^2^, which is an average of 4.3% (range 0.8–9.6%) from the area of the whole glenoid fossa.

**Table 1:** Dimensions of the tubercle of Assaky

Parameter	Mean ± standard deviation (mm/mm^2^)	Range (mm)
Height	0.9±0.2	0.1–1.0
Width	7.1 ± 2.3	3.9–21.1
Length	4.4±1.3	2.4–9.1
Area	29.5 ± 13.2	5.1–72.7

**Fig. 3:**
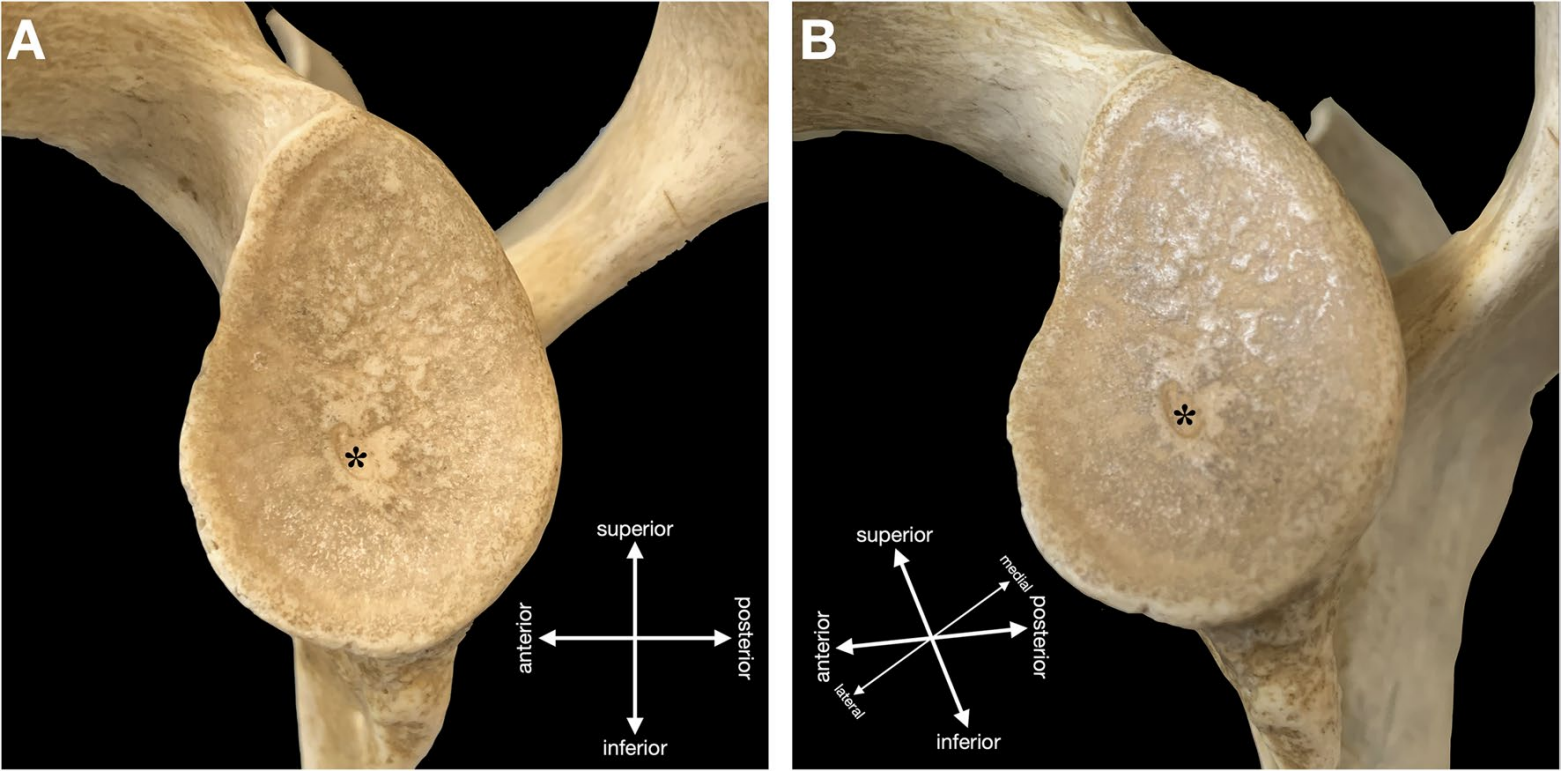
Left adult scapula containing an osseous depression (asterisk) inside the glenoid fossa. Captured from lateral (**A**) and posterolateral (**B**) views

Moreover, in 27 adult scapulae (9.0%), a centrally located depression was observed (Fig. 3). It was present in 12 right scapulae (8.1%) and in 15 left scapulae (9.9%) without statistically significant difference between the sides (*p* = 0.645). The dimensions of this depression were also recorded and are presented in Table 2. In comparison with the tubercle of Assaky, the depression occupied smaller area (13.8 ± 13.4 mm^2^) with an average of 1.9% (range 0.4–5.1%) from the area of the whole glenoid fossa. None of the scapulae featured the tubercle of Assaky and the central depression at the same time.

**Table 2:** Dimensions of the osseous depression

Parameter	Mean±standard deviation (mm/mm^2^)	Range (mm)
Depth	0.2±0.3	0.1–0.4
Width	4.0±2.4	1.7–6.4
Length	3.4±1.4	0.8–5.6
Area	13.8±13.4	2.2–33.7

**Fig. 4:**
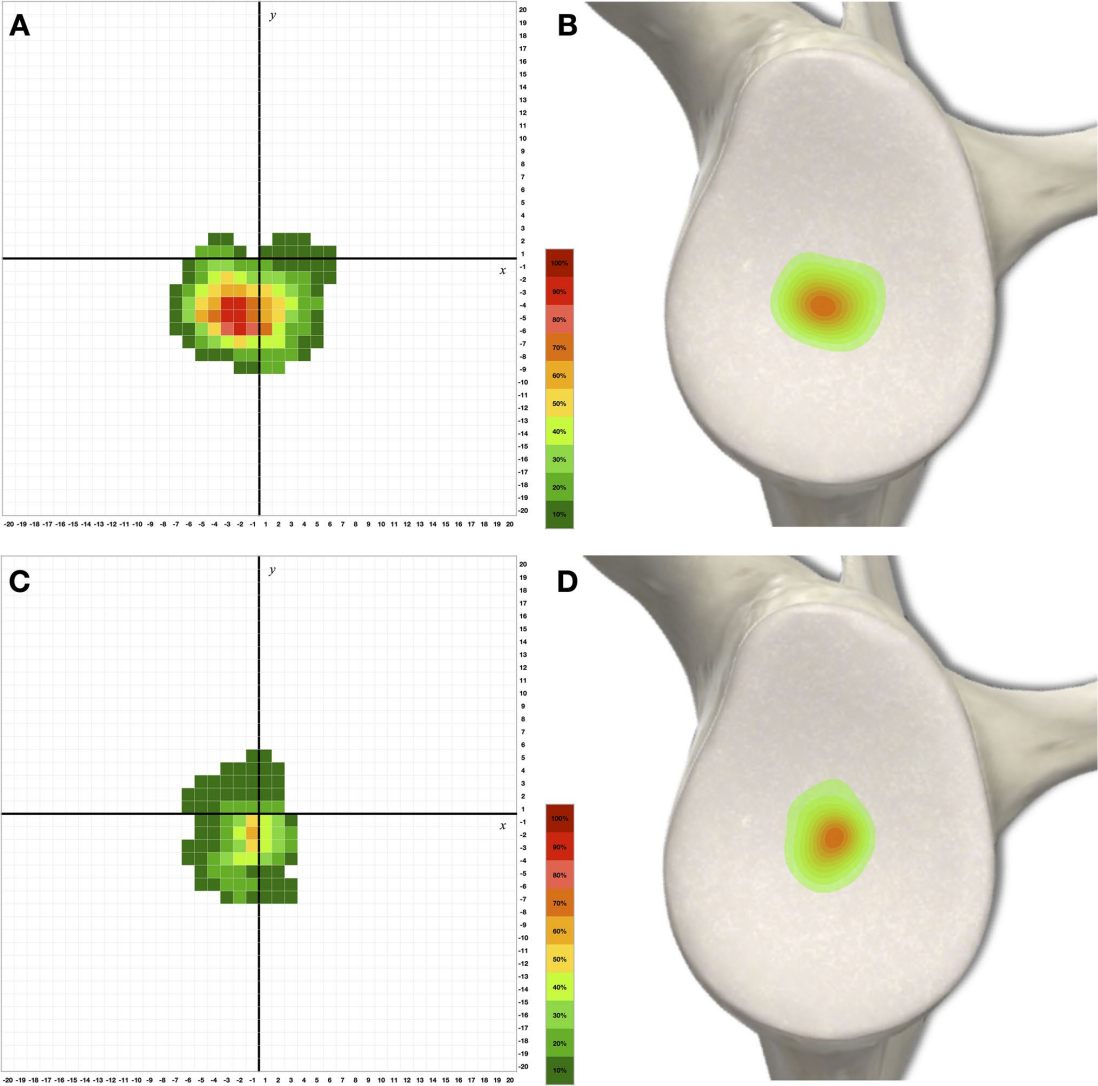
Constructed Cartesian coordinate system in a plane for the exact location of the tubercle of Assaky (**A**) and the osseous depression (**C**) with artistic presentation as heatmaps (**B**, **D**). Although only the left scapula is shown, results for the right scapulae using inverse values on *x*-axis were also included

The exact coordinates of the registered extent of the tubercle of Assaky and the bony depression were transferred into a unified Cartesian plane and graphically expressed as a heatmap. The tubercle of Assaky was located predominantly in the anterior-inferior quadrant of the glenoid fossa (Fig. 4A, B). Conversely, the depression tends to be located near the centre of the glenoid fossa (Fig. 4C, D).

**Fig. 5:**
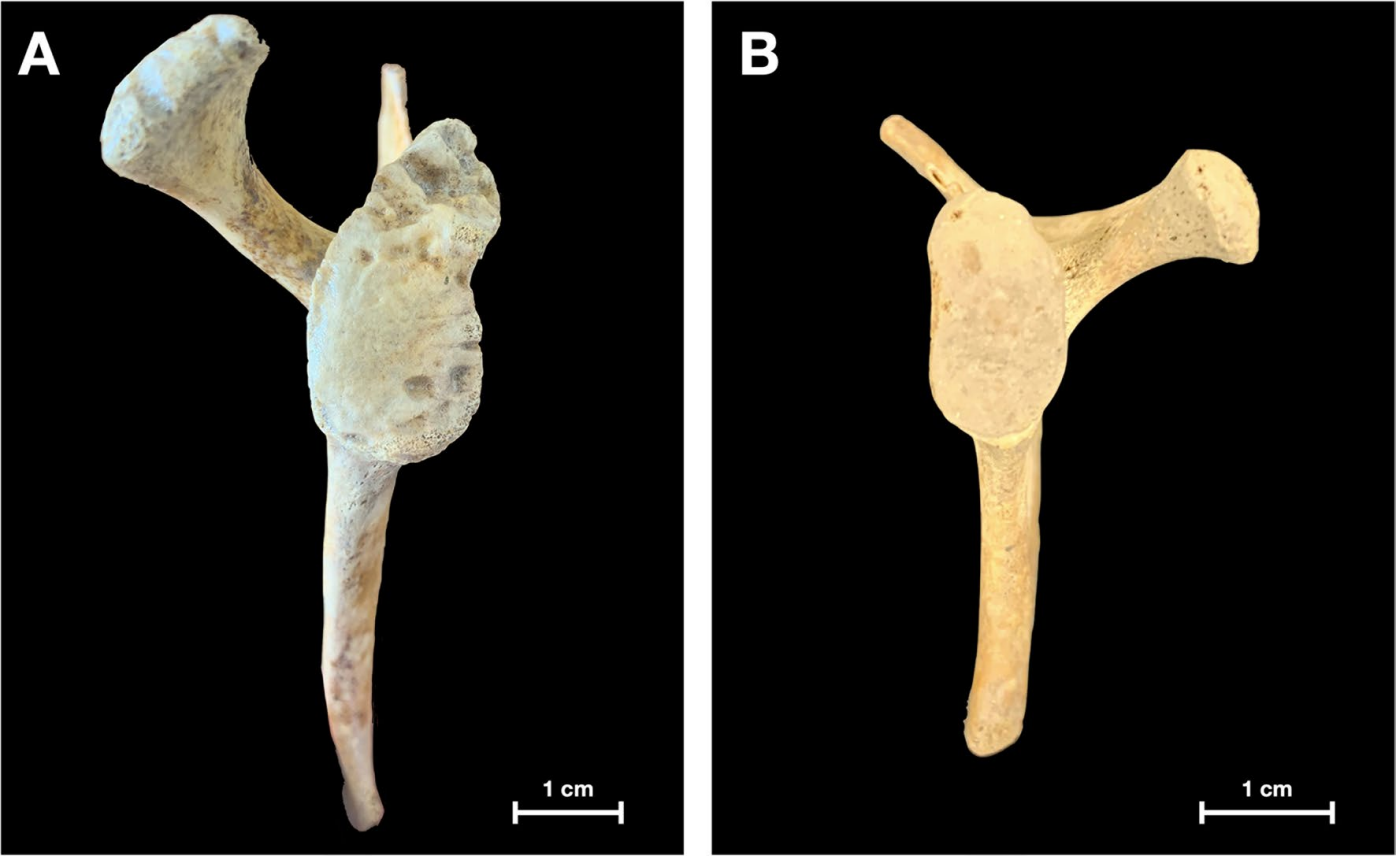
Children (**A**) and fetal (**B**) scapulae with focus on the glenoid fossa. Unlike the adult specimens, non-ossified glenoid and coracoid process can be seen

Neither the tubercle of Assaky nor the depression were detected in any of the children (0%), or fetal scapulae (0%). In all observed cases, the glenoid fossa was not fully ossified; therefore, these particular variations must have been developed after the age of 10 years (Fig. 5).

During a retrospective inspection of the photographed scapulae, we have noticed a frequent coincidence of a type III (severe) glenoid notch, according to the classification by Alashkham et al. [22], and the presence of the tubercle of Assaky (Fig. 2C). This purely morphological classification comprises three types, which are based on the depth of the glenoid notch. Out of the 140 scapulae with the tubercle of Assaky, 95 cases had the type III (severe) glenoid notch, 35 cases had the type II (moderate) glenoid notch and 10 of the observed cases featured the type I (mild) glenoid notch. The association of the tubercle of Assaky with the type III glenoid notch was statically evaluated as significant (*p* < 0.001).

### Radiological part

**Fig. 6:**
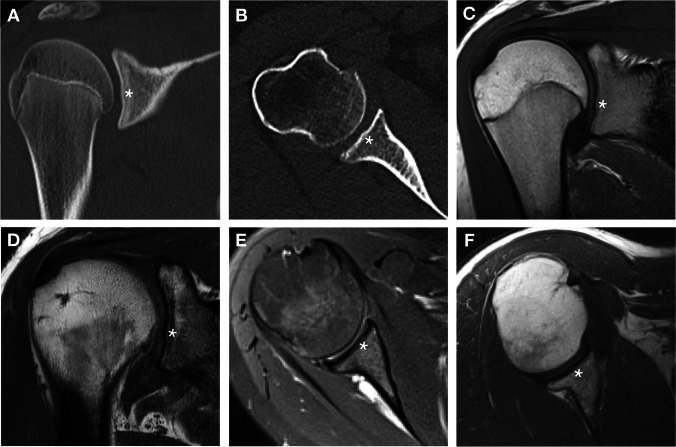
The appearance of the tubercle of Assaky (asterisk) on CT and MRI scans. CTs showing coronal slice of a 15-year-old male (**A**) and axial slice of a 69-year-old female (**B**). MRIs showing coronal slices of a 17-year-old female (**C**) and 31-year-old male (**D**); and axial slices of a 30-year-old female (**E**) and 52-year-old male (**F**)

The tubercle of Assaky was identified in 128 (42.7%) CT and in 118 (39.3%) MRI scans (Fig. 6). The tubercle was significantly found more often in male CTs (*p* < 0.001); however, MRIs did not show any statistically significant discrepancy between the genders. A higher occurrence on the right side was evaluated as significant in MRIs (*p* = 0.025), but these findings were not confirmed on CTs. Detailed findings regarding the side and gender distribution can be found in Table 3. Apparent thinning of the articular cartilage overlaying the tubercle of Assaky was observed in all cases (Fig. 6C–F).

**Table 3:** Detailed results showing the side and gender distribution of the tubercle of Assaky and the osseous depression detected on the CT and MRI arthrograms

Structure	CT						MRI					
	Right	Left	*p*	Male	Female	*p*	Right	Left	*p*	Male	Female	*p*
Tubercle of Assaky	55 (35.9%)	73 (49.7%)	0.259	98 (49.5%)	30 (29.4%)	< 0.001*	76 (42.7%)	42 (34.4%)	0.025*	70 (38.3%)	48 (41%)	0.150
Osseous depression	4 (2.6%)	8 (5.4%)	0.408	9 (4.5%)	3 (2.9%)	0.206	9 (5.1%)	5 (4.1%)	0.445	8 (4.4%)	6 (5.1%)	0.704

Percentages are calculated from the total number of the relevant side or gender

^*^Statistically significant difference

**Fig. 7:**
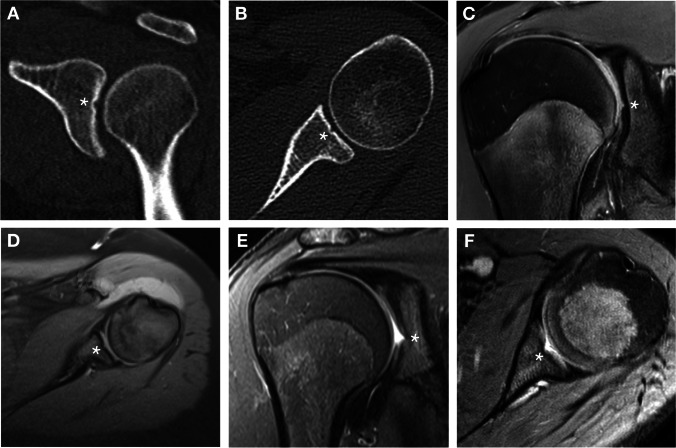
The appearance of the osseous depression (asterisk) on CT and MRI scans. CTs showing coronal and axial slices of a 25-year-old female (**A**, **B**). MRIs displaying the osseous depression covered with articular cartilage in coronal and axial slices of a 17-year-old male (**C**) and a 16-year-old female (**D**), respectively. MRIs showing a fluid-filled osseous depression in coronal and axial slices of two 15-year-old males (**E**, **F**)

In 12 (4.0%) CT and in 14 (4.7%) MRI scans, an osseous defect was recognised corresponding to the osseous depression on dry scapulae (Fig. 7). Neither the gender differences nor laterality unevenness were evaluated as statistically significant. On MRI examinations, the osseous depression was found either covered with the articular cartilage (11 cases; 78.6%) (Fig. 7C, D) or fluid-filled with complete absence of the cartilage (three cases; 21.4%) (Fig. 7E, F). A complete absence of the cartilage was identified only in patients of ages 15, 16 and 18. In all other cases, the articular cartilage was thinned above the rims of the osseous depression (Fig. 7C, D).

**Fig. 8:**
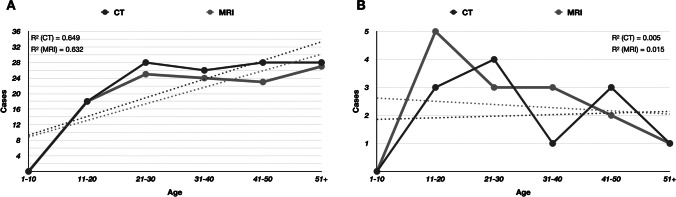
Plots showing the number of observed cases of the tubercle of Assaky (**A**) and the osseous depression (**B**) among different age groups. Dotted lines represent the linear graph

Appraisal of the different age groups yielded an increasing occurrence of the tubercle of Assaky among patients aged more than 10 years, and the dependency of presence with ageing was statistically confirmed (Fig. 8A). Nonetheless, the osseous depression was most frequently found in patients aged between 11 and 20 years. Visually, a decreasing incidence in older patients was noted, but *R*^2^ coefficient of determination was critically low (Fig. 8B).

### Arthroscopic part

**Fig. 9:**
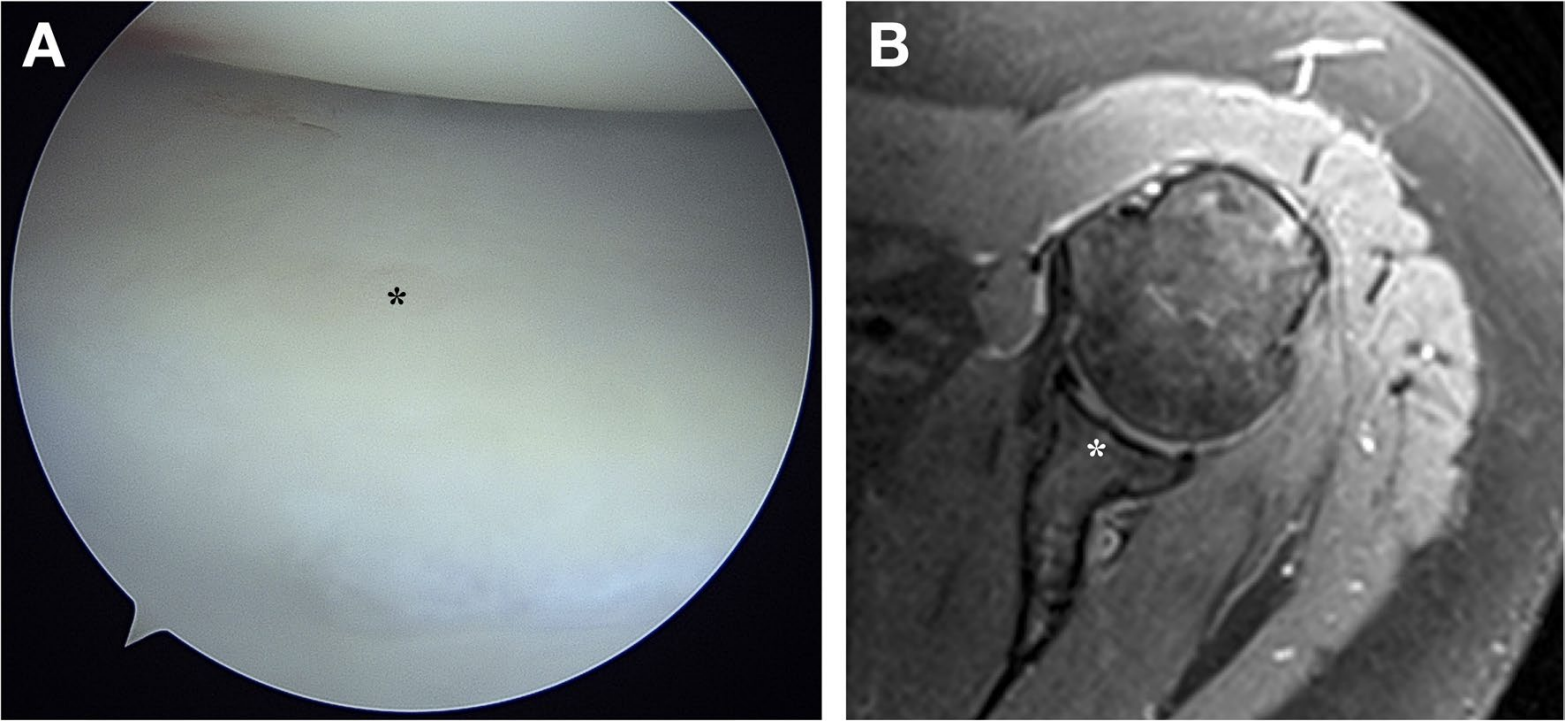
Arthroscopic view on a centrally located greyish area (asterisk) corresponding to the zone of thinned articular cartilage (**A**) and the MRI showing the underlaying tubercle of Assaky (asterisk) (**B**)

In 11 (55.0%) arthroscopies, a greyish area located near the centre of the glenoid fossa was identified (Fig. 9A). It was found in four (57.1%) males and seven (53.8%) females, without statistically significant difference between the genders (*p* = 0.366). The appearance of the grey spot was correlated with the preoperative MRI, where it corresponded to the thinned articular cartilage overlaying the tubercle of Assaky (Fig. 9B).

### Terminology consensus

Overall, 25 experts contributed to the construction of the newly established terms. Among these, 14 were orthopaedic surgeons (J.B., R.B., R.C., L.D.W., X.A.D., M.H., E.I., S.L., B.M., K.N., P.P., M.S., S.T., J.P.V.), 8 were anatomists (A.A., P.C., F.D., Q.F., O.N., S.S., T.T., A.V.) and 3 were radiologists (U.A., H.G., C.M.). Nonetheless, several orthopaedic surgeons are also active anatomists, and vice versa. All nominees consented to be publicly acknowledged and are alphabetically listed in the Acknowledgements section.

The goals of the Delphi method were to unanimously name the following four structures identified in our anatomical, radiological and arthroscopic observations:

- An osseous protrusion located approximately in the middle of the glenoid fossa consistent with the previously reported tubercle of Assaky (Fig. 1).
- An osseous depression located approximately in the middle of the glenoid fossa (Fig. 3).
- A macroscopically visual articular cartilage thinning overlaying the two abovementioned osseous variations (Fig. 9A).
- A complete absence of the articular cartilage due to the presence of the abovementioned osseous depression (Fig. 7E, F).

The first round of the survey contained four terms listed in Table 4. After the responses were received, two terms scored > 3.5 on average and were therefore finally selected. These are the glenoid fovea (*fovea glenoidalis*) for the osseous depression and the bare area of glenoid (*area nuda glenoidalis*) for the absence of the articular cartilage due to the glenoid fovea. The remaining two terms scored neutral grades. Therefore, adjustments and corrections were made based on the feedback and the survey for the second round was sent again to the same nominees.

**Table 4:** Detailed results of the Delphi consensus survey showing terms selection in both rounds

Term	1	2	3	4	5	Average score	Outcome
1st round (25/25—100% response rate)							
Glenoid tubercle (*tuberculum glenoidale*)	4 (16.0%)	3 (12.0%)	5 (20.0%)	6 (24.0%)	7 (28.0%)	3.36	Neutral
Glenoid fovea (*fovea glenoidalis*)*	3 (12.0%)	2 (8.0%)	2 (8.0%)	7 (28.0%)	11 (44.0%)	3.84	Concur
Gray macule of glenoid (*macula grisea glenoidalis*)	9 (36.0%)	3 (12.0%)	4 (16.0%)	4 (16.0%)	5 (20.0%)	2.72	Neutral
Bare area of glenoid (*area nuda glenoidalis*)*	2 (8.0%)	3 (12.0%)	2 (8.0%)	6 (24.0%)	12 (48.0%)	3.92	Concur
2nd round (21/25—84% response rate)							
Glenoid tubercle (*tuberculum glenoidale*)	7 (33.3%)	5 (23.8%)	1 (4.8%)	4 (19.0%)	4 (19.0%)	2.67	Neutral
Intraglenoid tubercle (*tuberculum intraglenoidale*)*	4 (19.0%)	3 (14.3%)	1 (4.8%)	4 (19.0%)	9 (42.9%)	3.52	Concur
Gray macule of glenoid (*macula grisea glenoidalis*)	12 (57.1%)	5 (23.8%)	1 (4.8%)	1 (4.8%)	2 (9.5%)	1.86	Oppose
Grey area of glenoid (*area grisea glenoidalis*)*	5 (23.8%)	0 (0%)	1 (4.8%)	8 (38.1%)	7 (33.3%)	3.57	Concur

^*^Definitively selected terms

The second round of the survey reached a consensus regarding the proposed terms for the two remaining structures. The term intraglenoid tubercle (*tuberculum intraglenoidale*) was selected as the systemic term for the tubercle of Assaky. Moreover, grey area of glenoid (*area grisea glenoidalis*) was selected to best describe the macroscopic cartilage thinning. All details of the electronic Delphi survey are shown in Table 4.

## Discussion

The key finding of the current study is that the central thinning of the glenoid articular cartilage is caused by the presence of the intraglenoid tubercle, as well as the glenoid fovea. In particular cases, the articular cartilage may be completely missing above the centre of the glenoid fovea. Both of these variations can be detected from the second decade of life. The incidence of the intraglenoid tubercle rises with aging, and the glenoid fovea is most commonly found in the second and third decades.

### Anatomical context and comparison with relevant literature

The former investigations were primarily addressed to the appearance of the articular cartilage. Considering the new nomenclature, the grey area of glenoid was found with a prevalence ranging from 48 to 100% within an adult population [1, 2, 6-9, 12, 13] and was not detected in any of the fetal specimens [18]. It must be mentioned that different methodological approaches were used across the reported studies for the assessment of the glenoid cartilage. In our arthroscopic observations, the grey area was with clear visualisation seen in 55.0% of cases. This is lower than expected, but our findings were very similar to those by Barcia et al. [7]. Conclusively, our findings highlight the inconstant presence of the grey area.

The intraglenoid tubercle as an independent structure is rarely discussed in the current literature. Indirect mention about its prevalence was found in an old anthropologic report informing about its presence in 40 out of 59 scapulae [23]. Therefore, the results of dry scapulae observations presented herein are more precise yielding an estimated prevalence for general adult population of 46.7%. Moreover, the intraglenoid tubercle was present in 42.7% CTs and in 39.3% MRIs comprising the whole age spectrum. In general context, laterality as well as gender tendencies were not sufficiently proven in our study.

A centrally located depression, termed the glenoid fovea, has been observed in two MRI studies of children population with a prevalence between 2.1 and 9.1% [14, 17]. In our investigation of adult scapulae, the glenoid fovea was present in 9.0% of cases. In correlation with the radiological imaging methods, using CT and MRI, the prevalence was 4.0% and 4.7%, respectively. These results must be carefully interpreted because different age groups were analysed. Laterality as well as gender distribution was statistically evaluated with no significant differences. We did not find the presence of the glenoid fovea and the intraglenoid tubercle in the same scapula; therefore, both variants appear independently.

During the arthroscopic observations, the location of the cartilage thinning was found above the intraglenoid tubercle, which was detected on the preoperatively performed MRI. Thus, we confirmed that the intraglenoid tubercle is responsible for the grey area of glenoid. Unfortunately, none of the treated patients in our study had the glenoid fovea. However, the thinning of the articular cartilage, or its eventual absence, adjacent to the glenoid fovea in arthroscopic images has been shown elsewhere [11, 24], so that conclusion can be drawn considering the glenoid fovea as a second morphological variant causing the grey area of glenoid, and in particular cases also the bare area of glenoid.

### Clinical relevance

Several researchers considered the bare spot (i.e. macroscopically visual cartilage thinning, newly named the grey area of glenoid) as a consistent landmark, which was nominated as a reference point for quantifying bone loss of the anterior glenoid rim during arthroscopy [1, 15]. Nevertheless, the former authors presumed that it is located at the centre of the inferior portion of the glenoid fossa. Aigner et al. [5], in their anatomical study, pointed out the significantly eccentric position of the bare spot making it an unreliable methodical point. These findings have been confirmed by other authors as well, and are also consistent with our results [6-9]. As the variant intraglenoid tubercle and the glenoid fovea appear in different locations projected on the glenoid fossa, the macroscopically visual grey area of glenoid features inconstant position depending on the underlying bony morphology. Consequently, the grey area is recommended for approximate perioperative assessment of the glenoid bone loss in shoulder instabilities, but it is unreliable for exact measurements [9]. A major limitation is also, as discussed above, its inconstant presence.

De Wilde et al. found the knowledge of the intraglenoid tubercle applicable in prosthetic reconstructions of the shoulder joint [16]. The bony protrusion should represent the strongest part of the glenoid bone which might be used for attainment of strong glenoid implant fixation. The fact that the tubercle overhangs the surface of the glenoid fossa, it must be borne in mind while reaming the bone for placement of the glenoid component to achieve a smooth and cohesive field. Apart from truly clinical applications, the intraglenoid tubercle was described as a constant anthropometric point for comparison of inter-individual differences between racial groups [23, 25].

Knowledge of the glenoid fovea is essential in preoperative evaluation of the glenoid defects. It should be distinguished from osteochondritis dissecans of the glenoid and glenolabral articular disruption (GLAD) lesions in the differential diagnostics. The clinical and radiologic features of these pathologies and the glenoid fovea were summarised by Ly et al. [11]. These conditions are diagnosed upon a thorough history, mechanism of injury, physical examinations and radiological findings—in particular, subchondral translucency, associated labral tear, associated osteochondritis dissecans elsewhere, presence of intraarticular bodies and associated Hill–Sachs lesions. Based on our findings, we highlight the specific inspection of the articular cartilage which is overlaying the fovea in majority of the cases (78.6%) and is therefore a suitable marker since it would not be present in the pathological conditions. Nevertheless, it must be carefully taken in mind that young patients may feature an absence of the overlaying cartilage, as demonstrated in 21.4% of our cases. A case of an imprecisely diagnosed and treated patient has already been reported in the literature [24].

### Developmental aspects

Together with the initial morphological report, Assaky also provided a technical note on the possible mechanism of the intraglenoid tubercle development [4]. He reported that the tubercle corresponds to the point of maximum pressure exerted by the head of the humerus and the articular cartilage is therefore narrowed by wear [4]. Later on, Testut questioned the ‘polar or juxta-central contact theory’ devised by Assaky [26]. According to his personal experience, based on sections of frozen subjects, he found that there is total contact between the glenoid fossa and the head of the humerus, which is ensured by a thin synovial layer [26]. Based on anatomical and histological study, DePalma believed that the tubercle develops around the fourth decade and is a form of degenerative process [27]. Nevertheless, contradictory ideas have been recently presented [14]. In contrast to what a study by Djebbar et al. has suggested, that the intraglenoid tubercle might be a remnant of the fusion between glenoid ossification centres [14], our results show that there is a mild discrepancy between the locations of the intraglenoid tubercle and the glenoid fovea, which is thought to develop due to the defective ossification. In particular, the incidence of the intraglenoid tubercle rises in older individuals. Thus, we suspect that the intraglenoid tubercle develops due to other reasons. According to our results, the tubercle emerges in the second decade and shows an increasing tendency with aging. Therefore, the role of a degenerative nature cannot be ruled out.

**Fig. 10:**
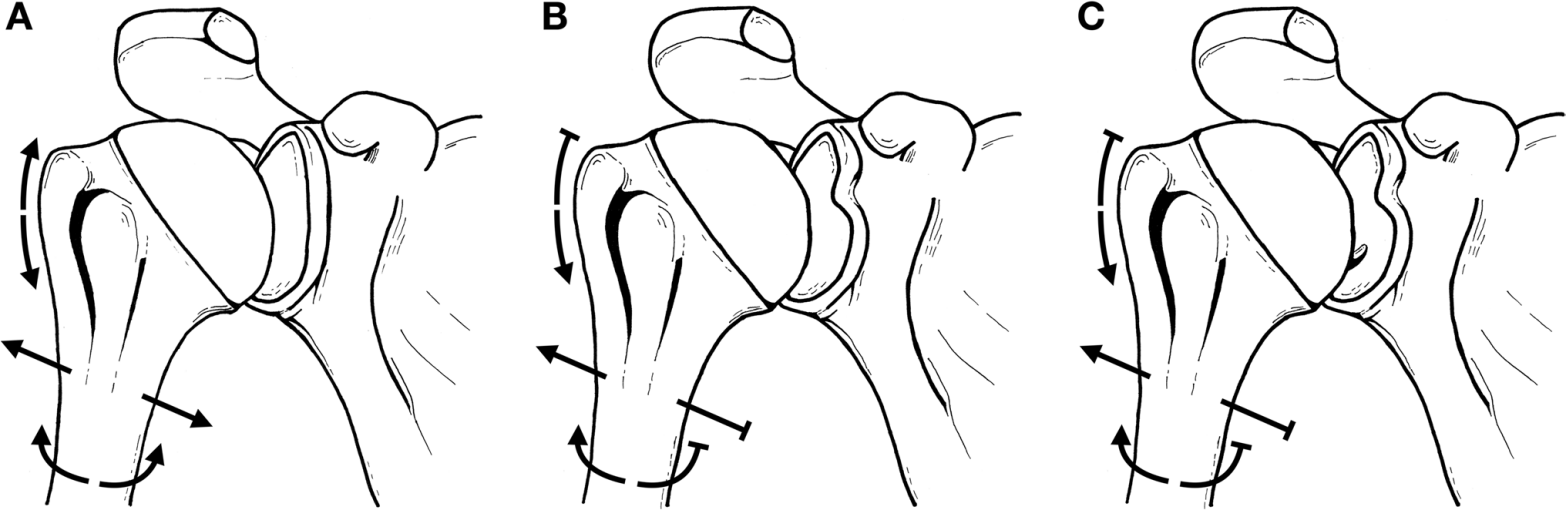
Schematic drawings illustrating the hypothesised mechanism of development of the tubercle of Assaky. Unlike in shoulders without the glenoid notch (**A**), the range of motion in shoulders with the glenoid notch may be slightly limited (**B**). Therefore, forces are centralised to a reduced area and, according to Wolff’s law [31], subchondral bone thickening occurs (**C**)

Possible explanation could be attributed to the reduction in size of the glenoid fossa either by presence of the glenoid notch or naturally thinner superior portion of the glenoid. The head of the humerus slides upwards or forward within the glenoid fossa while abducting or flexing and internally rotating the arm, respectively [28, 29]. Taking into consideration that the glenoid notch reduces the overall surface area of the glenoid fossa and forms a boundary at the rim of its extent, the range of translation of the head of the humerus is limited. Thus, repeated activity of the shoulder, especially abduction, flexion and internal rotation, of the shoulder centralises the forces to a minimised area that bears higher load in comparison with the remaining cartilage. Therefore, in correspondence with Wolff’s law, a reactive thickening and resurfacing of the subchondral bone occurs alongside the thinning of the overlaying cartilage [30, 31]. As a consequence, this remodelling process is assumed to form the tubercle at the location of the excessive contact (Fig. 10). To this theory also adds the eventual presence of the sickle type of the intraglenoid tubercle, which has a concave shape on its superior border conditioned to the convex head of the humerus.

**Fig. 11:**
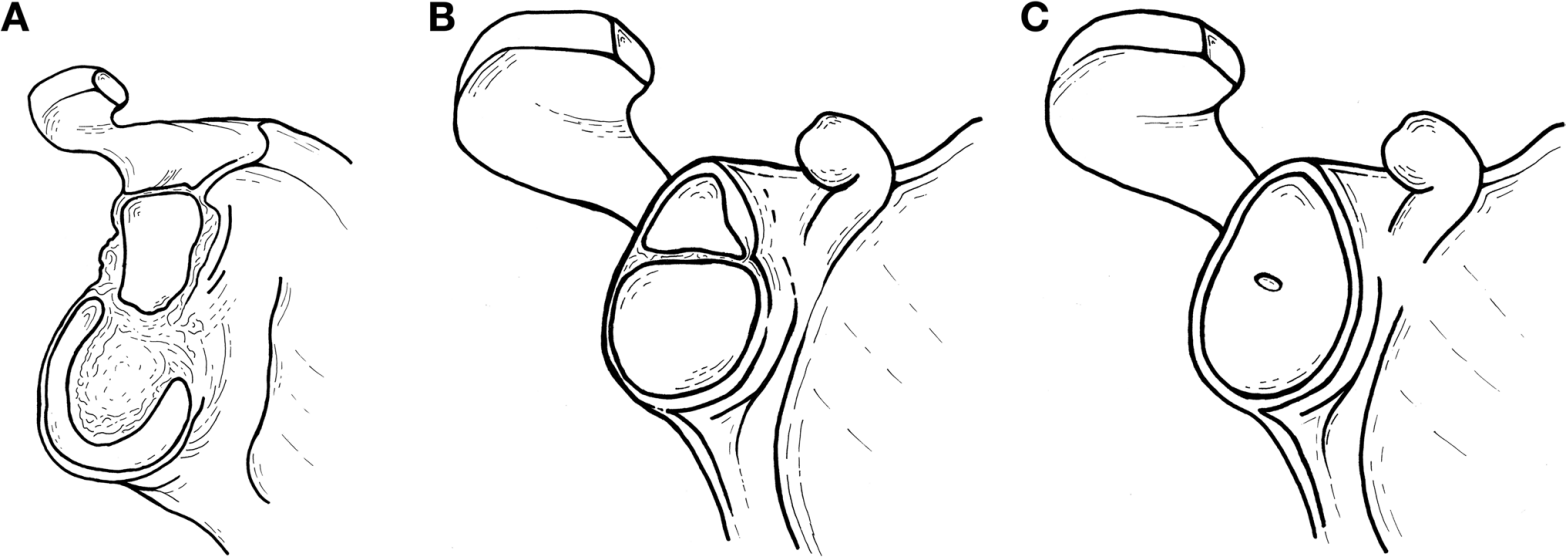
Schematic drawings illustrating the anticipated mechanism of development of the glenoid fovea. The subcoracoid ossification centre is responsible for the formation of the superior glenoid, while the inferior portion is formed by the horseshoe-shaped epiphysis derived from several small ossification islands (**A**). As these ossification centres approach each other to merge (**B**), a small defect may persist approximately in the middle of the glenoid fossa (**C**)

Precise mapping of the glenoid development is fundamental for understanding the formation of the glenoid fovea. Djebbar et al. postulated that the presence of the glenoid fovea is related to the usual pattern of ossification of the glenoid fossa [14]. Usually, the subcoracoid ossification centre is responsible for the formation of the superior portion of the glenoid and its ossification is completed by the age of 16 or 17 [32]. The inferior two-thirds of the glenoid are formed by the secondary ossification centres around the glenoid rim. Individual small ossification islands then merge to form a horseshoe-shaped epiphysis, which fuses with the periphery of the glenoid, and especially with the subcoracoid ossification centre. Complete fusion of the epiphysis with the remaining glenoid articular surface occurs between 17 and 18 years [32]. A slightly higher prevalence of the glenoid fovea was detected in the second decade, when the aforementioned ossification of the glenoid takes place. This is probably due to the incomplete fusion between the subcoracoid ossification centre and the epiphysis, or even between the small peripheral centres forming the rim. However, in some cases, there remains a small defect even after reaching the skeletal maturity that is most likely a result of defective ossification (Fig. 11). Taking into account the young age of patients with the fluid-filled glenoid fovea, we presume that the absence of the articular cartilage is only a temporary condition appearing before the closure of the ossification centres. Even if incomplete closure occurs, leading to the definitive formation of the glenoid fovea, the cartilage fills the created cavity over time.

### Historical perspective and terminological inconsistencies

The primary source describing the presence of a tubercle inside the glenoid fossa dates back to 1885 [4]. The description was presented by Romanian physician George Assaky [4]. Although Assaky was born in Romania, he moved to France after completing his high school studies to continue his academic career [33]; therefore, several authors addressed him as a French anatomist [30]. His elaborating work on a 4–5-mm-wide and approximately 1-mm protruding glenoid tubercle was presented on June 6th, 1885 at the session of the *Société de biologie* and subsequently published in their memoires [4]. This publication probably brought confusion about the spelling of Assaky’s name because right next to the title of the aforementioned article appears the name ‘G. Assaki’. However, in the introduction of the corresponding chapter as well as in the content section is written the name ‘G. Assaky’ [4]. This controversy presumably led future researchers to calling this osseous structure the ‘tubercle of Assaki’, which was afterwards generally accepted and is nowadays used in modern medicine [6, 7, 9, 10, 14, 16]. Several authors also used the term ‘tubercle of Asskay’ that is most likely a result of misspelling [11, 30].

To the best of our knowledge, there is no study focusing on a centrally located osseous depression inside the glenoid fossa in the currently published literature. Only Graves mentioned that in the centre of the glenoid, one will note a slight depression or elevation but did not comment on it further [25]. Although small attention has been given to its presence on children’s MRIs [14, 17], the glenoid fovea appears as a relatively new structure to be further studied.

As shown above, the anatomical terminology can be inaccurate and misleading. To unify and clarify the nomenclature regarding the structures discussed herein, we contribute with terminological proposals based on an expert consensus. The general term bare spot should be abandoned since it covers multiple individual structures. Instead, we suggest using the following four systemic terms. First, the ‘intraglenoid tubercle’ (*tuberculum intraglenoidale*) should substitute for the term tubercle of Assaky. The updated term is thought to best depict the macroscopic texture and the relationship with the glenoid fossa. Second, since there is no systemic term for the central osseous depression, the new term ‘glenoid fovea’ (*fovea glenoidalis*) has been invented. The Latin word fovea (pit) was agreed to best describe its macroscopic appearance on dry scapulae as well as on imaging methods. Third, an arthroscopically important structure representing a visual thinning of the articular cartilage was named the ‘grey area of glenoid’ (*area grisea glenoidalis*). This term refers to its greyish appearance denoted by historical researchers as well as current orthopaedic surgeons [26]. Lastly, the term ‘bare area of glenoid’ (*area nuda glenoidalis*) has been reserved for the complete absence of the articular cartilage due to the presence of glenoid fovea in teenagers.

This was a cross-sectional observational study with limited longitudinal data. In particular, we correlated the osteological observations with radiological and arthroscopic findings, each of which was obtained from different individuals. As such, it was not a consecutive series of diagnostics in one particular patient. Future long-lasting prospective studies could utterly confirm the developmental theories presented herein. Measuring by imaging methods was not obtained due to a challenging nature of the slices, which did not give measurable sectional views on the glenoid, particularly on the specific variants. Also, we used bony specimens with unknown sex, so we could not obtain data on possible gender-associated differences for comparison with the radiological imaging. However, the presented study underlines the irreplaceable role of osteological observations in translational research, which was found essential for understanding the background behind the cartilage irregularities. Unfortunately, the arthroscopic evaluations were performed on a limited number of patients. This may have influenced the number of present grey areas detected in our study, which was lower than what was reported elsewhere, but still within a similar range.

In conclusion, physiological articular cartilage thinning occurs due to the presence of the intraglenoid tubercle or the glenoid fovea. Although the intraglenoid tubercle is deemed as a clinically silent structure, the potential presence of the glenoid fovea must be carefully evaluated while interpreting osteochondral defects involving the glenoid fossa. Special attention requires interpretation of chondral defects in paediatric population because the cartilage may be naturally absent due to a glenoid fovea encompassing unclosed ossification centres of the glenoid. Moreover, the terminological updates are proposed to be implemented in clinical practice to obtain accurate descriptions of physiological processes concerning the glenohumeral joint.
